# Computed Tomographic Features of Benign and Malignant External Ear Canal Neoplasms in 39 Dogs

**DOI:** 10.1111/vru.70128

**Published:** 2026-01-09

**Authors:** Kaylynn Veitch, Christine Gremillion, Gwendolyn Levine, Cambridge Coy, Megan Wisnoski, John F. Griffin, Kenneth Waller

**Affiliations:** ^1^ Department of Surgical Sciences University of Wisconsin—Madison Madison Wisconsin USA; ^2^ Department of Large Animal Clinical Sciences College of Veterinary Medicine and Biomedical Sciences Texas A&M University College Station Texas USA

**Keywords:** computed tomography, external ear canal neoplasia, otitis externa

## Abstract

Computed tomography (CT) is commonly used to evaluate external and middle ear disease and for surgical planning in dogs. However, there is limited literature regarding CT characteristics of benign and malignant canine external ear canal neoplasms. This retrospective, multicenter, secondary analysis, cross‐sectional study compared the CT features of benign and malignant tumors in 39 dogs with 41 external ear canal masses by consensus of two veterinary radiologists. Recorded parameters were the presence of focal or multifocal tissue enlargement (mass/masses), lesion shape, location of the center of the mass, attenuation characteristics, features of contrast enhancement, involvement of otic structures, calvarial and brain changes, changes of nearby structures, and lymphadenopathy. The most common neoplasms in this study were ceruminous gland adenocarcinoma (13/41) and ceruminous gland adenoma (11/41). Although malignant tumors more commonly exhibited heterogeneous attenuation, heterogeneous contrast enhancement, aggressive periosteal proliferation, and compressed/displaced and/or invaded regional structures, benign tumors also exhibited aggressive characteristics, such as adjacent osteolysis. Given the degree of overlap of CT findings between benign and malignant external ear canal neoplasms, features may only aid in prioritizing differential diagnoses, and biopsy is required for definitive diagnosis.

## 1 Introduction

Computed tomography (CT) is widely used to diagnose middle ear disease in dogs and cats. CT is more sensitive and specific for diagnosing moderate to severe middle ear pathology compared to conventional radiography [1, 2]. Indications for CT of the ear canal in dogs include chronic otitis externa with treatment failure, external ear canal mass, neurologic signs, and surgical planning for total ear canal ablation and bulla osteotomy (TECA‐BO). Multiple papers have described detailed magnetic resonance imaging and computed tomographic normal anatomy of the canine external and middle ears [3, 4, 5, 6, 7, 8].

Aural neoplasms in dogs tend to be less biologically aggressive compared to those in cats [9]. Multiple studies have described computed tomographic findings of dogs with benign external and middle ear lesions, such as otitis externa, otitis media, inflammatory polyps, and cholesteatomas/tympanokeratomas [10, 11, 12]. Tumors arising from the middle ear in the dog are rare and are more commonly seen as an extension from the external ear canal [13, 14]. About 40% of tumors arising from the external ear canal are benign, including papilloma, sebaceous gland adenoma, ceruminous gland adenoma, and basal cell tumors. The most common malignant tumors arising from the external ear canal in dogs include ceruminous gland adenocarcinoma and squamous cell carcinoma [14]. Concurrent infection is common due to the obstructive nature of the tumor, leading to impaired drainage and accumulation of cerumen and debris, often complicating diagnosis and treatment. Ceruminous gland adenocarcinoma is commonly thought to develop secondary to chronic local inflammation, ceruminous gland hyperplasia, and increased exposure to cerumen [15, 16].

CT is commonly used to evaluate the presence and extent of external ear canal masses in dogs as well as surgical planning for potential TECA‐BO. To the authors’ knowledge, there are limited studies describing computed tomographic features of external ear canal neoplasms in dogs and no studies describing or comparing CT characteristics of multiple types of external ear canal neoplasms with a histopathologic diagnosis [17, 18]. Prior studies only describe differences in CT findings between otitis externa and external ear canal masses. No studies have described CT features of a specific external ear canal neoplasm or compared CT features between different tumor types of the external ear. Understanding specific CT characteristics of external ear canal neoplasia can help prioritize differentials to guide additional diagnostic tests and therapeutic intervention. Additionally, if there are distinct CT features of certain external ear canal neoplasms, this may be useful to aid in surgical planning, such as guiding decision‐making for the need for a bulla osteotomy, resection margin planning, or regional lymph node extirpation, and to help guide post‐imaging sampling of lesions. The purpose of this study was to describe computed tomographic findings of canine external ear canal neoplasia. A multicenter, retrospective study was concepted to collect a large cohort of canine cases of common external ear canal tumor types (both benign and malignant). An additional purpose of this study was to identify distinct CT features between ear canal tumor types.

## 2 Materials and Methods

This was a multicenter, retrospective, secondary analysis, cross‐sectional case series design. Medical records at Texas A&M University (TAMU) and the University of Wisconsin—Madison (UW) were searched for client‐owned dogs with histopathologically confirmed neoplasia of the external ear canal. Dogs were predominantly searched by the occurrence of a TECO‐BO surgical procedure. Dogs were included in this study if they had a contrast‐enhanced CT scan within 30 days of histopathologic diagnosis. Informed consent for use of clinical data was obtained from owners of all animals in accordance with institutional guidelines for client‐owned animals. As this was a retrospective study, no ethical approval was required.

Clinical data from medical records were collected by licensed veterinarians (C.C. and K.V.) and a third‐year veterinary student (M.W.). The following data were recorded: contributing institution, age (years), sex (male, castrated male, female, spayed female), breed, body weight (kilograms), side of the lesion (left or right), date of CT, recumbency during CT scan, whether the patient was sedated or anesthetized for the CT, date of surgery, clinical signs, and histopathologic diagnosis.

CT scans were performed on various helical CT systems per each institution's protocols. Slice thickness varied between 1 and 2.5 mm, pitch between 0.55 and 1, mA between 91 and 440, and kVp was consistent at 120. The scan field of view varied from 96 to 277 mm. At TAMU, matrix size varied between 512 × 512 and 512 × 1299 mm^2^. At UW, matrix size was consistent at 512 × 512 mm^2^. CT studies were reconstructed using bone and soft tissue algorithms. All CT studies were reviewed by two ACVR‐certified radiologists (C.G. and G.L.), and findings were recorded by a third‐year veterinary student (M.W.) based on consensus using a data collection form (Table S1). Images were evaluated using DICOM viewing software programs (eUnity 7.0 and IntelliSpace). Evaluators reviewed the entire study for all cases, and, at a minimum, all cases were evaluated in soft tissue and bone algorithms in the transverse plane. Evaluators were permitted to adjust the window width and window level. When available, additional reconstructions were reviewed (e.g., sagittal and dorsal plane reformats), or multiplanar reconstructions were evaluated. Evaluators were aware that the final diagnosis was external ear canal neoplasia but were unaware of the specific type of neoplasia and its location. Evaluators did not have access to the imaging reports for the case and did not review information other than the images provided. Interpretations were recorded, instances of initial discrepancy between reviewers were promptly discussed, and a final designation was reached by consensus agreement.

A mass was defined as the subjective perception of tissue enlargement. The number of masses and the side where tissue enlargement was the most severe were recorded. In short, masses were evaluated for shape (round/oval broad‐based vs. round/oval pedunculated vs. plaque‐like vs. amorphous), location (vertical ear canal, horizontal ear canal, middle ear, inner ear), attenuation characteristics, features of contrast enhancement (diffusely heterogeneous vs. heterogeneous with peripheral enhancement vs. homogeneous vs. none), involvement of otic structures (displacement, compression, invasion, destruction), calvarial and brain changes (periosteal proliferation, osteolysis, foraminal enlargement, meningeal thickening and enhancement, and intracranial invasion), and changes of nearby structures (lymph nodes, parotid salivary gland, skeletal muscle, and pharynx). Osteolysis was defined as the destruction of normal cortical or trabecular bone architecture. Amorphous, sunburst, spiculated, and palisading periosteal proliferations were considered aggressive. Smooth and lamellar periosteal proliferations were considered nonaggressive.

## 3 Results

### 3.1 Cases

The study population consisted of 42 dogs. Two cases were excluded from the study because the TECO‐BO surgical procedure and histopathology were performed >30 days after the CT scan. An additional case was excluded because the patient had bilateral external ear canal masses, and histopathological diagnosis was unavailable for one of the masses. The final study population consisted of 39 dogs from TAMU (*n* = 26, 67%) and UW (*n* = 13, 33%). Computed tomographic imaging was performed between March 2007 and March 2023.

### 3.2 Demographics

The study population had an age range between 2 and 16 years of age with a median age of 10 years. Weight ranged between 4 and 46 kg with a median of 13.8 kg. There were 6 male, 19 male castrated, 1 female, and 13 female spayed dogs. The most prevalent dog breeds included cocker spaniels (*n* = 8, 21%), Shih Tzu (*n* = 5, 13%), mixed breed dog (*n* = 5, 13%), Labrador retriever (*n* = 4, 10%), and dachshund (*n* = 2, 5%). A total of 41 external ear canal tumors were recorded. There were 19 (46%) tumors on the right side and 22 (54%) tumors on the left side. Two dogs had bilateral tumors. In one dog, both left and right external ear canal tumors were mixed adenomas. In the other dog, both left and right external ear canal tumors were ceruminous gland adenocarcinomas.

### 3.3 Tumor Types

**FIGURE 1:**
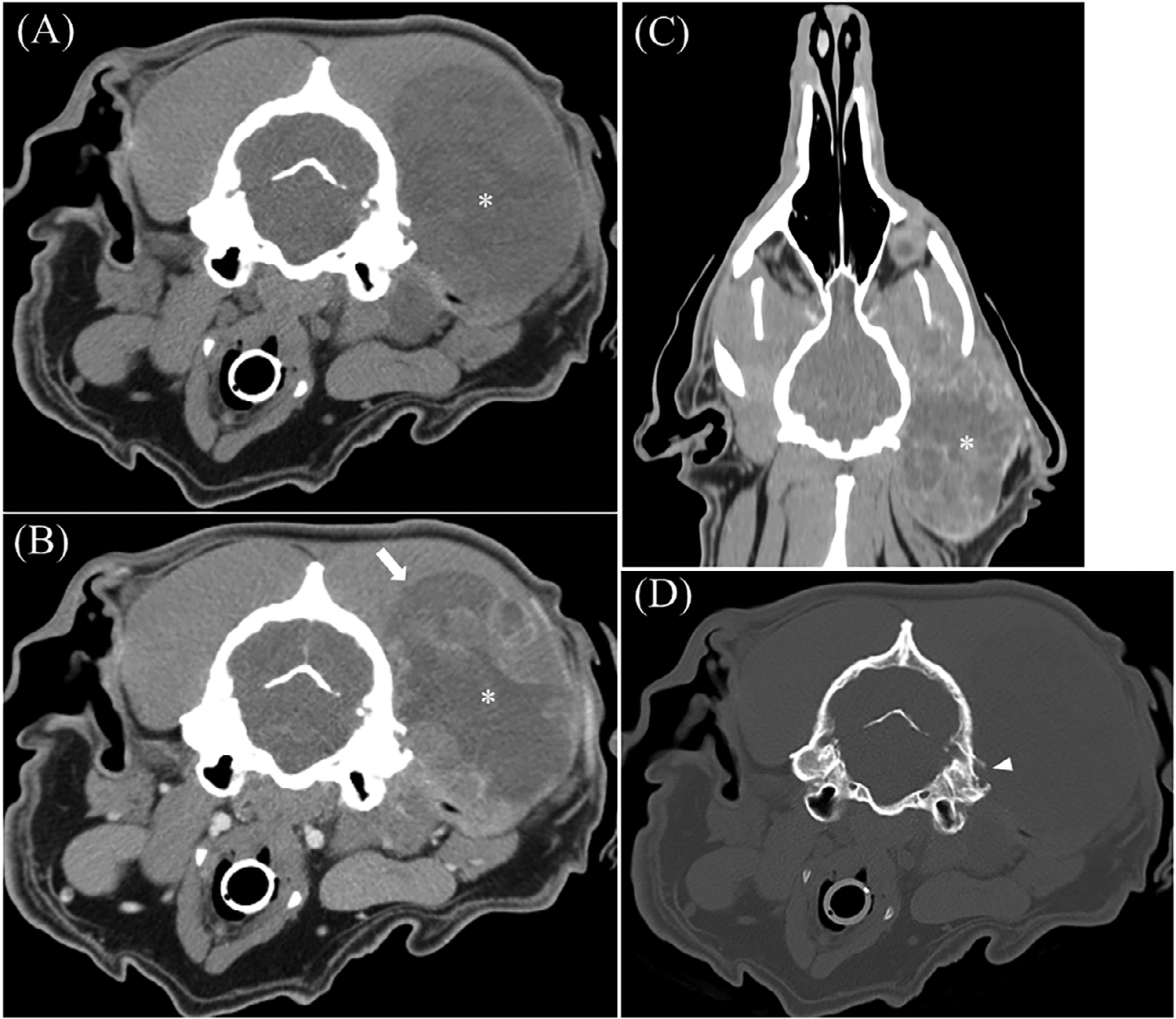
Pre‐contrast (A) and post‐contrast (B) transverse images, post‐contrast dorsal plane reconstruction (C) in a soft tissue algorithm (window width (WW) and window level (WL) of 400 and 40 Hounsfield units [HU], respectively), and (D) transverse image in a bone algorithm (WW/WL 2000/300 HU) in a 2‐year‐old male intact Labrador retriever with undifferentiated sarcoma centered on the left horizontal ear canal. This mass (asterisk) is heterogeneously soft tissue attenuating with intralesional fluid and is diffusely heterogeneously contrast‐enhancing. This mass invades into the left temporalis muscle (arrow). There is also calvarial osteolysis and aggressive periosteal proliferation of the mastoid process and supramastoid crest of the temporal bone (arrowhead).

The most common external ear canal tumors in this study were ceruminous neoplasms, including ceruminous adenoma (*n* = 11, 27%) and ceruminous adenocarcinoma (*n* = 13, 32%). Other benign neoplastic tumor types (*n* = 20, 49%) included plasmacytoma (*n* = 3, 7%), sebaceous adenoma (*n* = 2, 5%), mixed adenoma (*n* = 2), apocrine ductular adenoma (*n* = 1), and sebaceous epithelioma (*n* = 1). Additional malignant tumor types included apocrine adenocarcinoma (*n* = 2), squamous cell carcinoma (*n* = 1), anaplastic carcinoma (*n* = 1), papillary basosquamous carcinoma (*n* = 1), sebaceous adenocarcinoma (*n* = 1), fibrosarcoma (*n* = 1), and undifferentiated sarcoma (*n* = 1; see Figure 1).

### 3.4 CT Findings

The CT findings and comparisons for malignant versus benign tumors and ceruminous adenoma versus ceruminous adenocarcinoma are summarized in Tables 1, 2, 3. All masses that could be identified on CT (*n* = 38, 93%) were soft tissue attenuating and contrast‐enhancing. In terms of location, 17 masses (41%) were in the vertical ear canal, 20 (49%) were in the horizontal ear canal, and 1 tumor could not be determined if it arose from the vertical or horizontal ear canal. The majority of the tumors exhibited features of otitis externa, including ipsilateral ear canal fluid (*n* = 32, 78%), ear canal debris (*n* = 22, 54%), ear canal mineralization (*n* = 39, 95%), and ear canal thickening (*n* = 33, 80%). Approximately half of the tumors exhibited ipsilateral ear canal stenosis (*n* = 18, 44%). Regional lymph nodes were enlarged in the majority of cases, affecting the parotid lymph nodes (*n* = 30, 73%), medial retropharyngeal lymph nodes (*n* = 38, 93%), and mandibular lymph nodes (*n* = 22, 54%).

**TABLE 1:** Computed tomography (CT) findings in dogs with benign and malignant neoplasia.

	Benign tumors (*n* = 19)	Malignant tumors (*n* = 22)
**Mass shape**		
Round/Ovoid	6 (32%)	11 (50%)
Plaque‐like	7 (54%)	3 (14%)
Amorphous		1 (5%)
Combination	3 (23%)	7 (32%)
No mass	3 (23%)	
**Location**		
Vertical ear canal	9 (47%)	8 (36%)
Horizontal ear canal	6 (32%)	14 (64%)
Unable to characterize	1 (5%)	
NA (no mass)	3 (16%)	
**Attenuation characteristics**		
Homogeneous	11 (58%)	12 (55%)
Heterogeneous	5 (26%)	10 (45%)
Intralesional mineral	2 (11%)	5 (23%)
Intralesional fluid	1 (5%)	3 (14%)
**Contrast enhancement**		
Homogeneous	5 (26%)	3 (14%)
Heterogeneous	11 (58%)	19 (86%)
Non‐enhancing		
**Bony changes**		
Ipsilateral tympanic bulla thickening	6 (32%)	7 (32%)
Ipsilateral tympanic bulla expansion	2 (11%)	2 (9%)
Tympanic bulla osteolysis	5 (26%)	6 (27%)
Calvarial osteolysis	2 (11%)	3 (14%)
Aggressive periosteal proliferation	1 (5%)	4 (18%)
Nonaggressive periosteal proliferation	1 (5%)	
**Ipsilateral ear canal changes**		
Ear canal fluid	14 (74%)	18 (82%)
Ear canal debris	13 (68%)	9 (41%)
Ear canal mineralization	17 (89%)	22 (100%)
Ear canal thickening	16 84%)	17 (77%)
Ear canal stenosis	9 (47%)	9 (41%)
**Pharynx**		
Compression/Displacement		2 (9%)
Invasion/Destruction		
Combination		
**Surrounding musculature**		
Compression/Displacement	1 (5%)	6 (27%)
Invasion/Destruction		1 (5%)
Atrophy		
Combination		1 (5%)
**Parotid salivary gland**		
Compression/Displacement	1 (5%)	5 (23%)
Invasion/Destruction		
Combination		1 (5%)

**TABLE 2:** Computed tomography (CT) findings in dogs with ceruminous adenoma and ceruminous adenocarcinoma.

	Ceruminous adenoma (*n* = 11)	Ceruminous adenocarcinoma (*n* = 13)
**Mass shape**		
Round/Ovoid	4 (36%)	8 (62%)
Plaque‐like	4 (36%)	1 (8%)
Amorphous		1 (8%)
Combination	2 (18%)	3 (23%)
No mass	1 (9%)	
**Location**		
Vertical ear canal	5 (45%)	5 (38%)
Horizontal ear canal	4 (36%)	8 (62%)
Middle ear		
Unable to characterize	1 (9%)	
NA (no mass)	1 (9%)	
**Attenuation characteristics**		
Homogeneous	7 (64%)	8 (62%)
Heterogeneous	3 (27%)	5 (38%)
Intralesional mineral	1 (9%)	3 (23%)
Intralesional fluid	1 (9%)	
**Contrast enhancement**		
Homogeneous	5 (45%)	2 (15%)
Heterogeneous	5 (45%)	11 (85%)
Non‐enhancing		
**Bony changes**		
Ipsilateral tympanic bulla thickening	2 (18%)	4 (31%)
Ipsilateral tympanic bulla expansion	1 (9%)	2 (15%)
Tympanic bulla osteolysis	3 (27%)	3 (23%)
Calvarial osteolysis	1 (9%)	1 (8%)
Aggressive periosteal proliferation		1 (8%)
Nonaggressive periosteal proliferation		
**Ipsilateral ear canal changes**		
Ear canal fluid	8 (73%)	12 (92%)
Ear canal debris	8 (73%)	5 (38%)
Ear canal mineralization	9 (82%)	13 (100%)
Ear canal thickening	9 (82%)	10 (77%)
Ear canal stenosis	5 (45%)	5 (38%)
**Pharynx**		
Compression/Displacement		1 (8%)
Invasion/Destruction		
Combination		
**Surrounding musculature**		
Compression/Displacement	1 (9%)	3 (23%)
Invasion/Destruction		
Atrophy		
Combination		
**Parotid salivary gland**		
Compression/Displacement	1 (9%)	2 (15%)
Invasion/Destruction		
Combination		1 (8%)

**TABLE 3:** Regional lymph node enlargement in dogs with benign and malignant external ear canal neoplasia.

	Benign tumors (*n* = 19)	Malignant tumors (*n* = 22)
**Parotid LN**		
Yes	15 (79%)	14 (64%)
No	3 (16%)	8 (36%)
Not identified or not included	1 (5%)	0
**Medial retropharyngeal LN**		
Yes	18 (95%)	18 (82%)
No	1 (5%)	4 (18%)
**Mandibular LN**		
Yes	10 (53%)	11 (50%
No	9 (47%)	10 (45%)
Not identified or not included	0	1 (5%)

**FIGURE 2:**
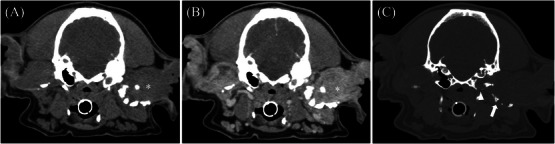
Pre‐contrast (A) and post‐contrast (B) transverse images in soft tissue algorithm (WW/WL 400/40 HU) and (C) transverse image in a bone algorithm (WW/WL 2000/300 HU) in a 12‐year‐old female spayed cocker spaniel with left ceruminous gland adenoma. This mass (asterisk) is homogeneously soft tissue attenuating and heterogeneously contrast‐enhancing. The mass involves and expands the horizontal and vertical external ear canals. Also note the ipsilateral tympanic bulla osteolysis (arrowhead) and severe mineralization of the auricles and annular cartilages of the left ear canal (arrow).

The ceruminous adenoma tumors (see Table 2 and Figure 2, mass identified in 10/11) were round/ovoid (*n* = 4, 36%), plaque‐like (*n* = 4, 36%), or a combination (*n* = 2, 18%) in shape and had mixed attenuation characteristics, being homogeneously attenuating (*n* = 7, 64%) and heterogeneously attenuating (*n* = 3, 27%) with contrast enhancement being homogeneous (*n* = 5, 45%) and heterogeneous (*n* = 5, 45%). Ipsilateral tympanic bulla thickening (*n* = 2, 18%), tympanic bulla expansion (*n* = 1, 9%), tympanic bulla osteolysis (*n* = 3, 27%), and calvarial osteolysis (*n* = 1, 9%) were identified in a subset of cases. No cases exhibited regional periosteal proliferation. The surrounding musculature and parotid salivary gland were displaced in one case.

**FIGURE 3:**
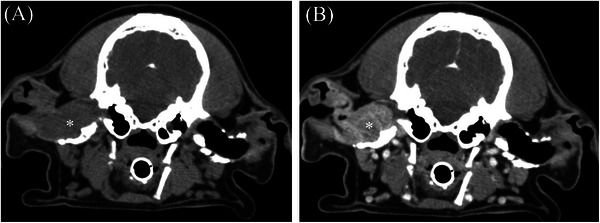
Pre‐contrast (A) and post‐contrast (B) transverse images in a soft tissue algorithm (WW/WL 400/40 HU) in a 9‐year‐old male castrated cocker spaniel with a ceruminous gland adenocarcinoma centered on the right horizontal ear canal. This mass is homogeneously soft tissue attenuating and heterogeneously contrast‐enhancing. This mass causes destruction of the horizontal ear canal. Note the severe mineralization and thickening of the external ear canals bilaterally.

The ceruminous adenocarcinoma tumors (*n* = 13; see Table 2 and Figure 3) were predominantly round/ovoid in shape (*n* = 8, 62%). The tumor attenuation was homogeneous (*n* = 8, 62%) and heterogeneous (*n* = 5, 38%), with intralesional mineralization in three (23%) tumors. The majority of the masses were heterogeneously contrast‐enhancing (*n* = 11, 85%). Ipsilateral tympanic bulla thickening (*n* = 4, 31%) and expansion (*n* = 1, 8%) as well as tympanic bulla osteolysis (*n* = 3, 23%) and calvarial osteolysis (*n* = 1, 8%) were identified in a subset of cases. Compression/displacement of surrounding structures, including the pharynx (*n* = 1, 8%), surrounding musculature (*n* = 3, 23%), and parotid salivary gland (*n* = 2, 15%) were also identified. One case exhibited parotid salivary gland invasion/destruction and compression/displacement.

As there was a wide variation in tumor types in this study, tumors were divided into benign (*n* = 19, 46%) and malignant (*n* = 22, 54%) groups. Notable distinguishing features (as seen in Tables 1 and 2) included attenuation characteristics and contrast enhancement. Benign tumors were more commonly homogeneous in attenuation (*n* = 11, 58%) and less commonly heterogeneous (*n* = 5, 26%). Malignant tumors exhibited homogeneous attenuation (*n* = 12, 55%) and heterogeneous attenuation (*n* = 10, 45%) with similar prevalence. Intralesional mineral (benign, *n* = 2; malignant, *n* = 5) and intralesional fluid (benign, *n* = 1; malignant, *n* = 3) were identified in both benign and malignant tumors. Contrast enhancement was variable, including homogeneous contrast enhancement (benign, *n* = 5; malignant, *n* = 3) and heterogeneous contrast enhancement (benign, *n* = 11; malignant, *n* = 19). A few of the malignant tumors affected adjacent structures, including compression/displacement of the pharynx (benign, *n* = 0; malignant, *n* = 2), compression/displacement and/or invasion of the surrounding musculature (benign, *n* = 1; malignant, *n* = 8), and compression/displacement and/or invasion of the parotid salivary gland (benign, *n* = 1; malignant *n* = 6).

## 4 Discussion

The purpose of this study was to characterize the CT features of histopathologically confirmed external ear canal neoplasia in dogs and identify distinctive CT features for discrimination between benign and malignant neoplasms. In this study, approximately 47% (20/42) of tumors were benign. This is in agreement with a previous report in which 40% of canine external ear canal tumors were benign. This is in contrast to the cat, where the majority of aural neoplasms are malignant, with some resources reporting up to 87% of cases [14, 19, 20]. The most common neoplasms in this study were ceruminous gland adenoma and ceruminous gland adenocarcinoma.

In three instances, a distinct mass on CT was not identified. All three of these masses were benign. One dog accounted for two of these instances, with a mixed adenoma being diagnosed in both the right and left external ear canals, but no mass was identified by the evaluators on CT. A mild (<50% of the horizontal ear canal) amount of fluid/debris was identified in the right external ear canal, but not the left. Severe auricular and annular cartilage mineralization and external ear canal thickening were noted in both ears, resulting in stenosis. The other dog without an observable mass on CT was diagnosed with a ceruminous adenoma. Lack of identification of a mass may be due to the small size of the mass and/or difficulty in distinguishing a mass from chronic inflammatory changes to the ear canal, such as thickening, folding, and contrast enhancement of the ear canal. The second dog that exhibited bilateral external ear canal neoplasia (ceruminous gland adenocarcinoma) had similar mass morphology and characteristics bilaterally, but a discrepancy in the severity of the masses. Both masses were described as “plaque‐like” or diffusely infiltrative along the vertical ear canal with homogeneous contrast enhancement, intralesional mineralization, and destruction of both the vertical and horizontal ear canals. The mass on the right side invaded into the middle ear with tympanic bulla expansion and mixed thickening and osteolysis, whereas the left‐sided mass did not show invasion or destruction of the middle ear. However, there was fluid/debris within the external ear canals and tympanic cavities on both sides. Both masses also caused compression/displacement of the parotid salivary glands, whereas only the right‐sided mass caused compression and possible invasion of the right masseter and temporalis muscles.

A few studies have described imaging characteristics of nonneoplastic aural lesions in dogs, including otitis externa and otitis media, tympanokeratomas (formerly known as cholesteatomas), and inflammatory polyps [1, 2, 10, 12, 18, 21, 22]. Few have described imaging CT characteristics of aural neoplasia in the dog [13, 18]. Our study found many common and overlapping CT findings between malignant and benign aural neoplasms. Overlapping characteristics included intralesional mineral, intralesional fluid, and adjacent bony changes such as ipsilateral tympanic bulla thickening and expansion, tympanic bulla osteolysis, and calvarial osteolysis. Chronic ear canal changes and regional lymphadenopathy were also common features of both groups. Aggressive characteristics, including tympanic bulla and calvarial osteolysis, and periosteal proliferation, are not unexpected findings for both benign and malignant lesions, as these dogs typically have concurrent chronic otitis externa and otitis media [15, 23]. This degree of overlap between benign and malignant neoplasia with concurrent chronic inflammatory ear canal changes further complicates a radiologist's ability to prioritize differentials.

However, a few overlapping features were slightly more common in the malignant group, including horizontal ear canal location, heterogeneous attenuation, heterogeneous contrast enhancement, and aggressive periosteal proliferation. In this case series, malignant tumors also more commonly compressed/displaced and/or invaded regional structures, including the pharynx, parotid salivary gland, and surrounding musculature. This is in line with previous studies, which report that malignant ear canal neoplasia may be locally invasive and best managed with aggressive surgical excision [19]. However, as expressed previously, primary or secondary infection can also result in aggressive changes and mass effect to surrounding tissues. Therefore, benign etiologies should remain high on the differential list. The relationship between mass size and the presence of bony pressure resorption or regional tissue compression/displacement was not assessed in this study. Biopsy and histopathology should always be performed for definitive diagnosis.

There were a few limitations to this study. As this was a retrospective study, complete medical records and case histories were not always available. Although many of these patients presented for chronic otitis externa, the duration of clinical signs and treatments attempted was not recorded or used in analysis. The effect of concurrent chronic otitis externa on the interpretation of ear canal and middle ear disease on CT is unknown. The evaluators in this study were partially blinded to the histopathological results. Although they were aware that the patient was diagnosed with external ear canal neoplasia, they were unaware of the specific tumor type and location. There is a possibility that this biased the evaluators during the CT evaluation. The survey the evaluators completed was designed to be objective to minimize this bias.

In this study, cases were required to have a histopathologic diagnosis and concurrent CT, and the majority of cases were searched by patients who had undergone TECA‐BO for severe, chronic otitis externa/media or neoplasia. This may have biased the population toward dogs with more severe and chronic disease. Patients may also have been referred for cross‐sectional imaging due to the severity of the patient's disease. If our patient population was biased toward dogs with more severe and chronic disease, this may explain why there was such a significant overlap in CT features between benign and malignant tumor types, as some of these tissue changes may be due to chronic otitis rather than direct tumor effects. Finally, the case numbers for each type of neoplasia in this study were relatively low. Due to the low case numbers, this study was descriptive in nature without statistical analysis. We hope that our results will inform future studies aiming to evaluate the predictive values of certain imaging features for the diagnosis of specific external ear canal neoplasms.

## 5 Conclusion

In this retrospective, multicenter cross‐sectional study, CT characteristics of dogs with different types of benign and malignant aural neoplasia overlapped. Even benign tumors exhibited aggressive characteristics, such as tympanic bulla and calvarial osteolysis. Malignant tumors more commonly exhibited heterogeneous attenuation, heterogeneous contrast enhancement, aggressive periosteal proliferation, and compressed/displaced and/or invaded regional structures (pharynx, parotid salivary gland, and surrounding musculature). Due to the degree of overlap in CT characteristics of benign and malignant external ear canal neoplasia, biopsy and histopathologic diagnosis are highly recommended in these cases.

## Disclosure

Findings from the present study have not been reported in a previous meeting or published in an abstract.

## Conflicts of Interest

The authors declare no conflicts of interest.

## Supporting information




**Supporting File**: vru70128‐sup‐0001‐DataCollectionForm.xlsx

## Data Availability

The data that support the findings of this study are available from the corresponding author upon reasonable request.
